# Chest compression rate measurement from smartphone video

**DOI:** 10.1186/s12938-016-0218-6

**Published:** 2016-08-11

**Authors:** Kjersti Engan, Thomas Hinna, Tom Ryen, Tonje S. Birkenes, Helge Myklebust

**Affiliations:** 1 Department of Electrical and Computer Engineering, University of Stavanger, Stavanger, Norway; 2 BI Builders, Sandnes, Norway; 3 Laerdal Medical, Stavanger, Norway

**Keywords:** Video processing, Motion analysis, Smartphone, CPR, FFT

## Abstract

**Background:**

Out-of-hospital cardiac arrest is a life threatening situation where the first person performing cardiopulmonary resuscitation (CPR) most often is a bystander without medical training. Some existing smartphone apps can call the emergency number and provide for example global positioning system (GPS) location like *Hjelp 113-GPS* App by the Norwegian air ambulance. We propose to extend functionality of such apps by using the built in camera in a smartphone to capture video of the CPR performed, primarily to estimate the duration and rate of the chest compression executed, if any.

**Methods:**

All calculations are done in real time, and both the caller and the dispatcher will receive the compression rate feedback when detected. The proposed algorithm is based on finding a dynamic region of interest in the video frames, and thereafter evaluating the power spectral density by computing the fast fourier transform over sliding windows. The power of the dominating frequencies is compared to the power of the frequency area of interest. The system is tested on different persons, male and female, in different scenarios addressing target compression rates, background disturbances, compression with mouth-to-mouth ventilation, various background illuminations and phone placements. All tests were done on a recording Laerdal manikin, providing true compression rates for comparison.

**Results:**

Overall, the algorithm is seen to be promising, and it manages a number of disturbances and light situations. For target rates at 110 cpm, as recommended during CPR, the mean error in compression rate (Standard dev. over tests in parentheses) is 3.6 (0.8) for short hair bystanders, and 8.7 (6.0) including medium and long haired bystanders.

**Conclusions:**

The presented method shows that it is feasible to detect the compression rate of chest compressions performed by a bystander by placing the smartphone close to the patient, and using the built-in camera combined with a video processing algorithm performed real-time on the device.

## Background

Out-of-hospital cardiac arrest (OHCA) is a major cause of mortality globally [1]. Arrested blood circulation prevents delivery of oxygen and glucose to the body, resulting in absent breathing and brain injury. Immediate treatment is important to increase chance of survival. There are around 400,000 OHCAs each year in the US alone, and only 10 % survive^1^. Most of these cardiac arrest situations will happen without the presence of medical professionals. Often the bystanders are friends or family of the patient, and are likely to get stressed by the situation. In case of OHCA, time to cardiopulmonary resuscitation (CPR) should be as short as possible and should continue with high quality until return of spontanous circulation. When bystanders perform CPR, quality can be variable and sometimes ineffective, even for health care professionals. In a study where ambulance performed the CPR, chest compressions were not delivered half of the time during CPR [2]. CPR feedback can improve CPR quality for both lay people and professionals [3–6]. Continuous coaching by a dispatcher can also improve CPR quality [7, 8] and is now widely available as most callers carry phones with a speaker function. Today almost everybody have a smartphone, permitting apps with functionality beyond hands-free verbal communication. Many apps are made to support the caller or dispatcher in case of cardiac arrest. Some provide GPS location and hands-free and simple dialing of the emergency number, like *Hjelp 113-GPS* App by the Norwegian air ambulance^2^ and *Emergency+* available on App store and Google play. Others, like *PulsePoint*, are carried by CPR volunteers, who will receive a notification from the dispatcher in case of nearby emergency and help the volunteer reach the victim [9], and can locate automated external defibrillators (AEDs) which can be dispatched to the scene. Other apps provide audio and visual coaching to help the bystander, and some uses the build-in accelerometer to guide CPR performance. The most recent guidelines on cardiopulmonary resuscitation state that the dispatcher plays a critical role in the provision of CPR. Currently, the dispatcher has no objective information about how CPR is performed [10].

The aim of this work is to present an algorithm that can be embedded in such apps utilizing the built in camera in the smartphone for doing automatic detection of chest compression rate, and communicating the chest compression rate to a dispatcher.

### Previous work

There are some studies and attempts on using the accelerometer in the phone to perform both compression rate and compression depth measurements [11–14]. One example is the *Zoll PocketCPR* app for Android and iOS^3^, a publicly available app. Using such an approach, the bystander has to hold the phone in the hand while doing compression. The phone needs to be held correctly, and it can possibly block the microphone and the loudspeaker. The bystander wastes time putting down and picking up phone when doing CPR 30:2 (30 compressions followed by two ventilations). The *Zoll PocketCPR* app is only in use for training CPR for the moment, and is not embedded in any emergency app.

A short correspondence by Frisch et al. [15] is presenting a trial performed on manikins using the camera in the smartphone for the detection of compression rate, showing some encouraging results. The correspondence lacks details, but suggests a method based on finding the difference between consecutive frames and extracting the repetitive motion as the compression rate. All data has ongoing compression activities with rates between 60 and 144 pr. minute, and the same placement of the camera. The detection algorithm was performed off-line in MATLAB.

To our knowledge there are no references where the smartphone camera has been used for detection of compression rate, where the calculation has been done in real time on the phone, which will be presented in this work. Also, our algorithm is tested in more situations and with more disturbances than in [15].

## Methods

**Fig. 1:**
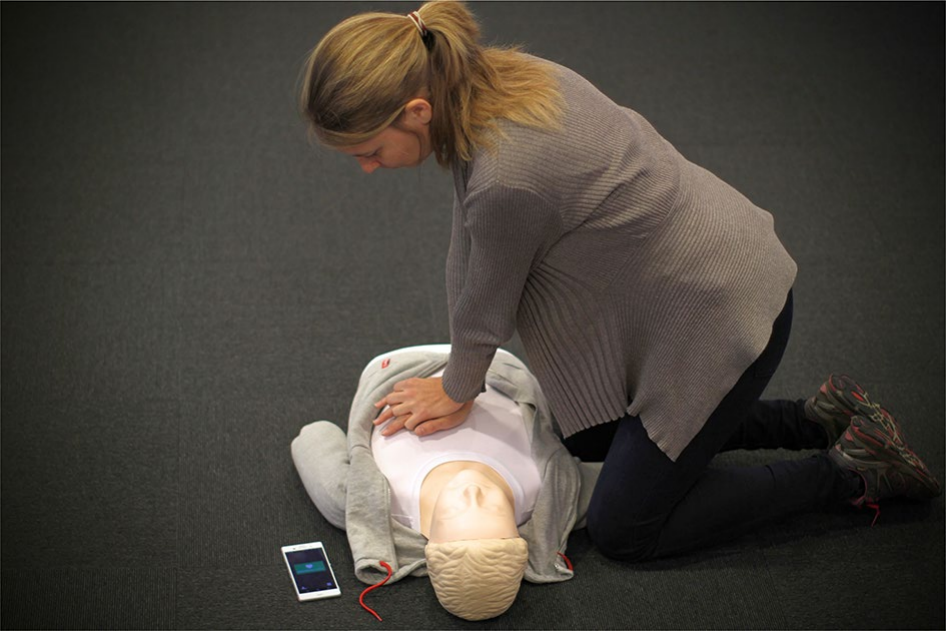
Illustration of CPR and placement of smartphone

In this paper, we present a method where the camera in a smartphone is activated by an emergency app, to provide the caller and dispatcher with information on the compression performance of the bystander. The dispatcher will first instruct the bystander to place the phone on the floor next to the head/chest of the patient, as illustrated in Fig. 1, and to start chest compressions. The recorded video is fed through an algorithm extracting the main frequency of a repetitive ongoing motion in the scope of the camera, and the detected compression rate is sent to the dispatcher in real time as extra information throughout the conversation. The algorithm takes into account that only part of the bystander motion will be captured on video, like just the shoulder moving in the corner of the display, or it can cover most of the frame. The bystander might stop doing compressions, other people moving in the background, the phone itself can be moved, or the bystander might change position.

**Fig. 2:**
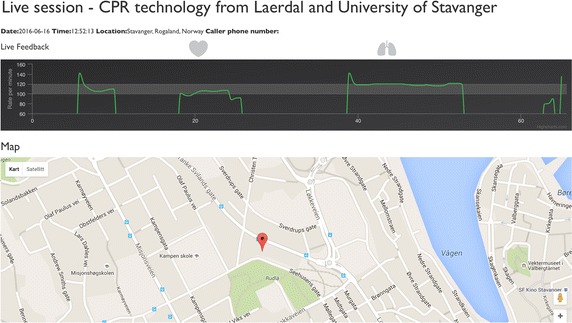
Example of live feedback given to the dispatcher. The *upper plot* shows the detected compression rate. The *grey field* of 100–120 cpm indicates the recommended compression rate. At the *bottom*, the GPS information from the phone is displayed

Figure 2 illustrates an example of the live feedback received by the dispatcher, where the GPS location of the phone is shown on a map, and the live feedback curve at the top shows the estimated compression rate as a function of time. The European Resuscitation Council (ERC) guidelines [10] states that the desired compression rate is 100–120 cpm. This desirable compression rate area is marked as a gray area on the generally black background of the compression plot, as seen in Fig. 2. In the example shown, we see there are pauses between periods of chest compression.

### Compression rate detection

**Fig. 3:**
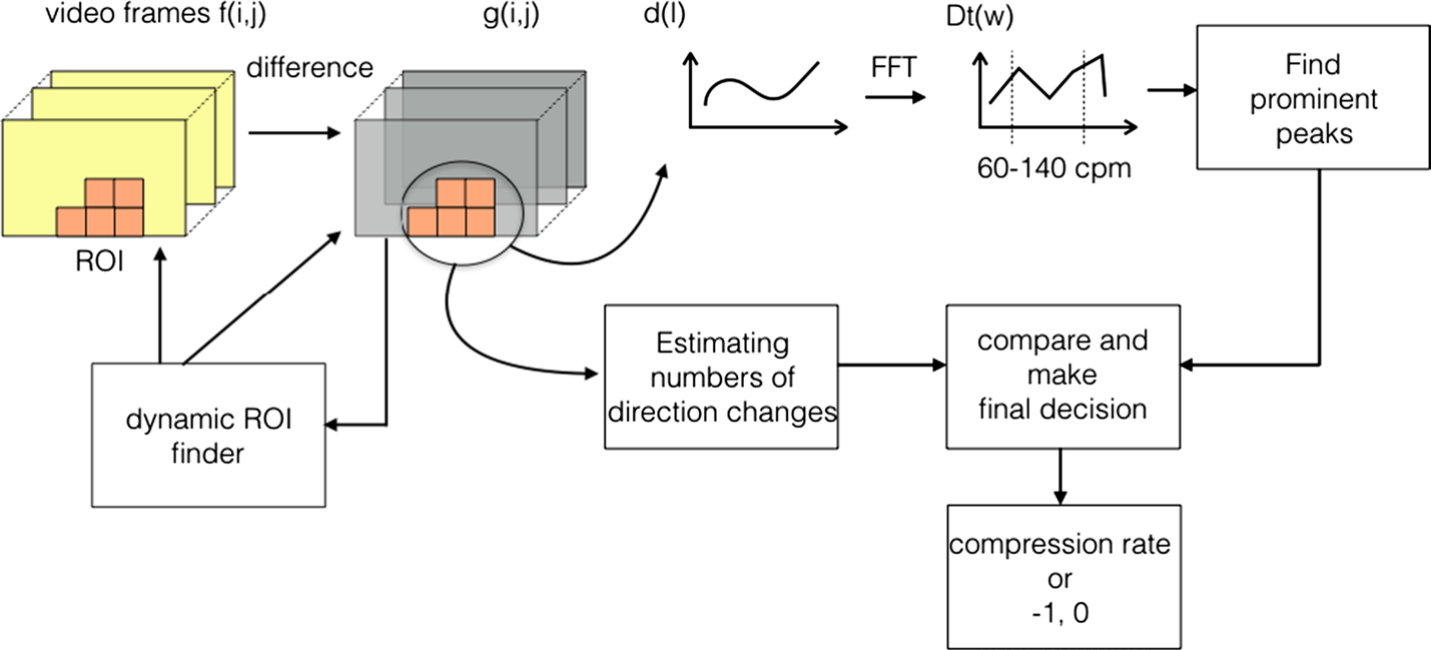
Simplified block scheme of the proposed system

In this section we will present the core ideas of the proposed algorithm, whereas in the next section some details of the implementation of the real time smartphone app is presented. In Fig. 3 a simplified block scheme of the proposed system is depicted. This will be described in the following.

Let $f_{l}(i,j)$ represent video frame number *l*, where (*i*, *j*) corresponds to row index *i* and column index *j*. For two consecutive image frames, define the difference image $g_{l}$ as:

1 $$\begin{aligned} g_{l}(i,j) = \left\{ \begin{array}{l l} 0, &{}\quad \text {if }\; |f_l(i,j) - f_{l-1}(i,j)| \leq \varepsilon \\ |f_{l}(i,j) - f_{l-1}(i,j)|, &{}\quad \text{otherwise} \end{array} \right. \end{aligned}$$

where $\varepsilon$ is a chosen threshold. The core idea is to use fast fourier transform (FFT) on the sum of the pixels in the difference image since the repetitive movement of chest compression should provide a peak in the FFT at the right frequency. Based on the placement of the phone relative to the bystander and patient the movement of the bystander might cover most of the video frame, or it might be that only a part of the shoulder is visible in a corner of it. To make the algorithm robust, a dynamic region of interest (ROI) scheme must be included. The algorithm has to be able to run in real time, thus accurate segmentation of the bystander is to computationally demanding to consider.

The difference image $g_{l}(i,j)$ is divided into non-overlapping blocks of size 50 × 50 pixels, $R_{k}$ ordered row by row.

2 $$\begin{aligned} S_{R_{k}}(l)=\sum _{(i,j)\in R_{k}}g_{l}(i,j) \end{aligned}$$

gives the sum of change in region block $R_{k}$ for time-point (frame number) *l*. When deciding which block should be part of the dynamic ROI, the changes over the last 15 frames, i.e. times step of 0.5 s when video rate is 30 fps, are considered at the time. When establishing a new ROI, four such consecutive time-steps are studied, i.e. 60 frames. Let

3 $$\begin{aligned} S^{L}_{R_{k}}(t)=\sum _{m=(t-1)L}^{tL}S_{R_{k}}(m)=\sum _{m=(t-1)L}^{tL} \sum _{(i,j)\in R_{k}}g_{m}(i,j) \end{aligned}$$

denote the sum of changes for block $R_{k}$ summed over the last $L=15$ difference frames, at time point *t*, where $l=t\cdot L$. Let $\bar{S}_{R}^{L}(t)$ denote the average of all the sums of the different region blocks.

For all blocks, $R_{k}$, and $L=15$, an indicator function is defined:

4 $$\begin{aligned} I_{R_{k}}(t) = \left\{ \begin{array}{l l} 1, \quad &{} S^{L}_{R_{k}}(t) > \bar{S}_{R}^{L}(t) \\ 0, \quad &{} else \end{array} \right. \end{aligned}$$

When establishing a new ROI, a specific block, $R_{k}$, is included in the ROI if at least three of the last four indicator values were one:

5 $$\begin{aligned} R_{k} \in \{ \text{ ROI } \} \;\; \text{ if } \sum _{n=t-3}^{t} I_{R_{k}}(n) \ge 3 \end{aligned}$$

After this the ROI is not necessarily one connected object, as we want it to be. A three-step procedure is followed: (1) Gaps in the ROI is filled. This might add some regions that should not have been added. (2) All blocks in the ROI with $\sum _{n=t-4}^{t} I_{R_{k}}(n)=0$ is removed. This might break the ROI up to multiple objects again. (3) Finally the largest of the connected objects (groups of blocks) in the ROI is chosen as ROI.

After the ROI is established, it can be updated and will be changed over time. If there at some point in time are no blocks in the ROI, it is re-established. During ROI updating, all blocks at the boundaries of the ROI are checked. Let $R_{bo_{i}}$ denote block *i* on the outside of the boundary, i.e. not already in the ROI, and $R_{bi_{i}}$ denote block *i* on the inside of the boundary, i.e. already inside ROI.

6 $$\begin{aligned} R_{bo_{i}} \in \{ \text{ ROI } \} \;\; \text{ if } \sum _{n=t-2}^{t} I_{R_{bo_{i}}}(n) = 3 \end{aligned}$$

7 $$\begin{aligned} R_{bi_{i}} \notin \{ \text{ ROI } \} \;\; \text{ if } \sum _{n=t-1}^{t} I_{R_{bi_{i}}}(n) = 0 \end{aligned}$$

One exception is if a $R_{bo_{i}}$ is at a corner, then it will be included when $\sum _{n=t-2}^{t} I_{R_{bo_{i}}}(n) = 2$.

When an ROI is established the difference signal at time point *l* is found using the sums defined in Eq. 2:

8 $$\begin{aligned} \text{ d }(l)=\sum _{R_{k}\in \text{ ROI }} S_{R_{k}}(l) \end{aligned}$$

Now we have a time signal *d*(*l*), calculated over a dynamic sized ROI. The number of blocks in the ROI is kept as $N_{b}(l)$. The ratio : $d_{ROI}=\frac{d(l)}{N_{b}(l)}$ is found and compared to a threshold; if $d_{ROI}<Th_{1}$ there is no/very litle activity going on and the compression rate, *CR*(*l*) is set to 0.

If $d_{ROI}>Th_{1}$, the FFT is performed on each block of $L_{f}=90$ values of the *d*(*l*) time signal, and $N_{b}(t)$ is constant for each block. The power spectrum density is estimated by the periodogram [16]:

9 $$\begin{aligned} D_{t}(w)=\frac{1}{L_{f}}|\mathcal {F}(d(j))|^2 \;\;\; j=(t-1)L_{f}:tL_{f} \end{aligned}$$

We wish to find the most dominant frequency in the video segment, but have to take into account that there might not be any compression activity, and that there might be other movements in the image frame. Thus all frequencies outside the area of 60–140 compressions pr. minute (cpm), are discarded, and let $w_{int}$ denote this frequency area of interest. Firstly the total energy of this frequency band over the number of blocks in ROI is found:

10 $$\begin{aligned} D_{ROI}=\sum _{w\in {w_{int}}}(D_{t}(w))^2 / N_{b}(t) \end{aligned}$$

If $D_{ROI}<Th_{2}$ then $CR(t) = -1$. The label −1 for the compression rate is used to label the rate as uncertain. If $D_{ROI}>Th_{2}$ the algorithms proceeds by finding the most dominating frequencies within $w_{int}$. The frequency corresponding to the maximum energy is found:

11 $$\begin{aligned} w_{m}={{\mathrm{argmax}}}_{w} D_{t}(w), \;\; w \in w_{int}, \end{aligned}$$

and all frequencies $\{w_{set}\}=w>(w_{m}\cdot Th_{3}), \; w \in w_{int},$ are investigated further. $Th_{3}$ is empirically set to 0.18. The prominence/distinction of $D_{t}(ws)$ with respect to it’s neighbors are evaluated: $P_{ws}={{\mathrm{prom}}}\,D_{t}(ws) \; ws \in \{w_{set}\}$, and if $P_{ws}<Th_{4}$, $ws \notin \{w_{set}\}$, i.e. the frequencies where the energy does not stand out from its neighboring frequencies are removed from the set $w_{set} \rightarrow w_{set_{1}}$. If the set becomes empty, i.e. $w_{set_{1}}=\emptyset , \rightarrow CR(t)=-1$.

For all $ws \in w_{set_{1}}$ check if also $2 {ws} \in w_{set_{1}}\rightarrow ws=ws^{'}$. This is because for a given compression rate we will often find a dominant peak at double the frequency as well. Thus the energy of these two frequencies are added together. Thereafter the values $D_{t}(ws)$ are compared to $D_{ROI}$, the energy in the interesting frequency band. If

12 $$\begin{aligned} \frac{D_{t}(ws)}{D_{ROI}}>Th_{4}+\delta \end{aligned}$$

$ws \in \{w_{set_{2}}\}$, if not it is removed. For $ws=ws^{'}, \delta \ne 0$. Else $\delta =0$. Now the final set $w_{set_{2}}$ is checked. If $w_{set_{2}}=\emptyset , \rightarrow CR(t)=-1$. If $w_{set_{2}}$ has only one frequency, $w_{final}$ this corresponds to the compression rate:

13 $$\begin{aligned} CR(t)=f(w_{final},\text{ fps},L_{f}). \end{aligned}$$

If $w_{set_{2}}$ has more than one frequency, they are compared to a frequency estimation using an alternative approach where the number of direction changes is estimated by looking at the difference images. If one of the frequencies from the different methods are (almost) the same, this frequency is chosen as $w_{final}$. If none of the potential frequencies corresponds to the alternative method; $CR(t)=-1$.

### Implementation

**Fig. 4:**
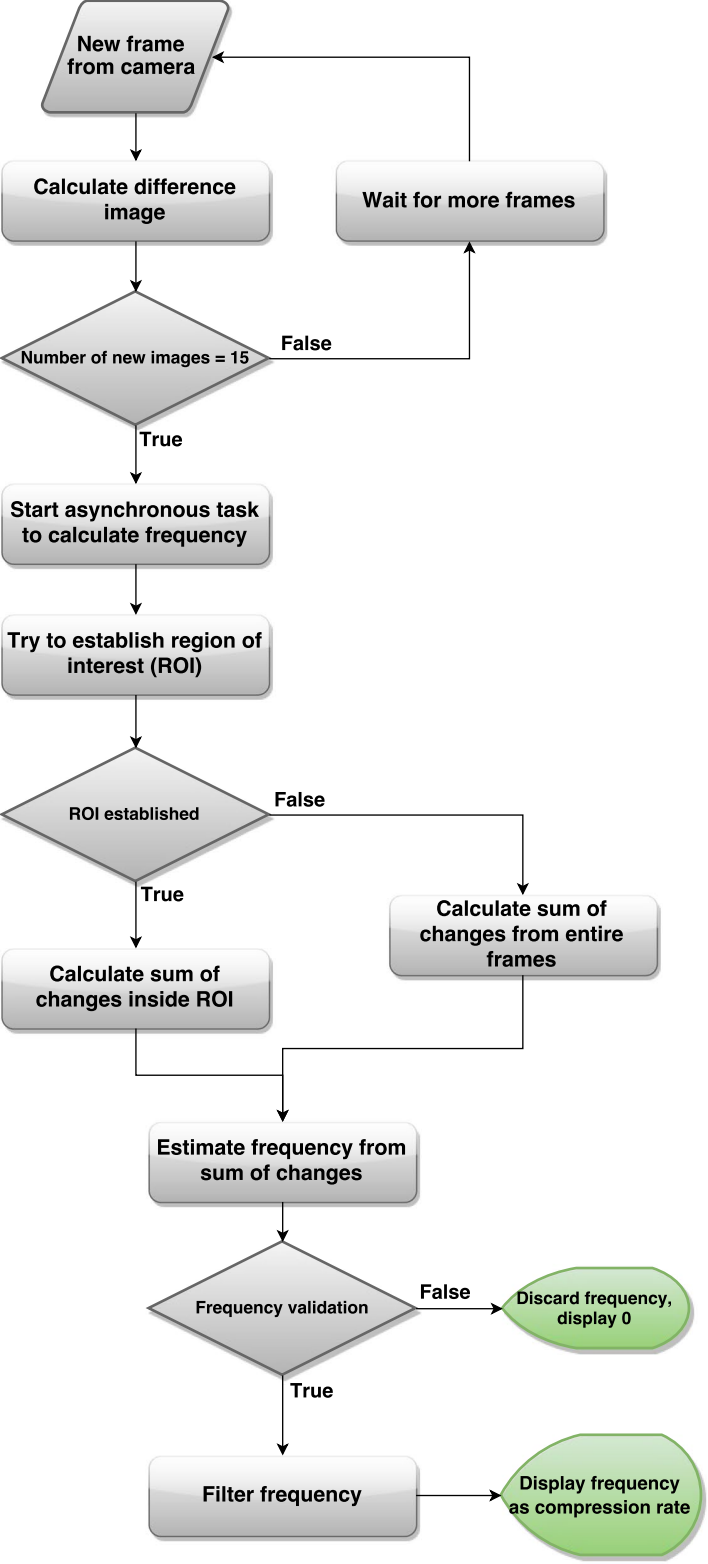
Algorithm flowchart

The proposed system is implemented and tested on a smartphone, and this section describes some of the implementation details. The algorithm is implemented as an Android application. It has primarily been tested on a LG Nexus 5, but short tests has also been done on a few other phones/tablets. It is built to support Android 4.1 (SDK version 16) and newer. A flowchart describing the algorithm can be seen in Fig. 4. All thresholds mentioned in the previous section, $\varepsilon , \; Th_{i} \; i=1\ldots 4$, are chosen empirically during pre-tests and are thereafter kept constant.

#### Getting the camera frames

The camera is instantiated with a resolution of 640 × 480 pixels and a frame rate of 30 fps (frames per second). This is done by getting a list of supported frame rates and resolutions from the camera and selecting the closest to the desired ones. This desired resolution and frame rate should be supported by most smartphones available today. Image data from the camera are available via the camera preview, the live camera feed usually displayed on the screen. A preview callback is attached to the camera instance to provide a callback every time a new frame is available. Each new frame is delivered in a byte array, in YCbCr color format. Only luminance value is used, i.e. color information is discarded to save computation time. Preliminary tests indicated that the results with or without color information was virtually identical.

#### Algorithm overview

Every time a new frame from the camera is available, the difference image between the new frame and the previous frame is calculated. When 15 difference images (every 0.5 s with 30 fps) has been calculated, an asynchronous task is started to estimate the compression rate, if compression is detected. An asynchronous task is a task that runs in the background. Here it is used to avoid lag in the user interface and to prevent frame drops. This task has the biggest workload of the algorithm. It uses the 15 new difference images provided, as well as up to 75 of the previously used (making it a total of up to 90 images, equal to 3 s with 30 fps). First, the task tries to establish an ROI. If an ROI can not be established yet, the whole frame is used as ROI. The sum of changes is calculated inside the ROI, using up to 90 of the previous images. We use FFT and some other techniques to estimate the dominating frequency in the window we are examining. This dominating frequency has to pass several tests before being trusted. If the frequency is not trusted it means the algorithm does not believe that it is caused by chest compressions, but by some other movement. If trusted, a weighted moving average filter is applied to the frequency, and thereafter it is transmitted and displayed as compression rate in cpm.

#### User interface

**Fig. 5:**
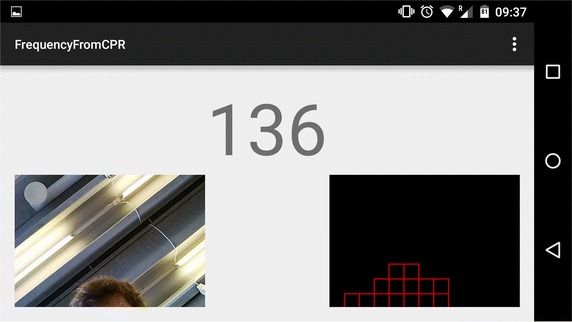
Screenshot of smartphone during use of the proposed app. Number corresponds to detected compression rate. *Left image* shows video frame, *red squares* in* right image* indicates region of interest

Figure 5 displays an example screenshot of the smartphone during use of the proposed app. The compression rate is displayed on the phone (here; the number 136) as well as transmitted to the dispatcher. The left image shows the video frames as captured by the phone, so that the bystander can see if the camera is catching his or her movement. The right image shows the dynamic ROI as the red squares. This will change and be updated over time.

### Experiments

A set of testing scenarios has been executed to test the performance of the implemented Android algorithm. The tests include consecutive compressions with various rates, CPR 30:2, background disturbances, suboptimal phone placement, variations in light conditions and a test for false positives. In total, there were nine different tests, which were all performed by seven test persons. The duration of each test is in the range of 60–120 s. The test persons were selected based on their hair length, to test the algorithm using various hair types. Long and loose hair will move a lot during compression, and the movement is more chaotic. Thus long and loose hair introduces substantial noise. A short description of each test person is seen in Table 1.

**Table 1:** Test persons used in the experiments

Test person	Description	Group
1	Man with short hair	Short
2	Woman with long and loose hair	Long
3	Woman with her hair up	Short
4	Man with short hair	Short
5	Woman with long and loose hair	Long
6	Woman with chin length hair	Medium
7	Woman with shoulder length hair	Medium

The experiments were performed by doing compression and CPR 30:2 on a Resusci Anne QCPR training manikin (Laerdal Medical, Norway).^4^ The Resusci Anne training device records the depth of compressions and the rate of compression as a function of time, and this rate is stored and considered reference data in these experiments. The smartphone app was running simultaneously recording the detected compression rates on the smartphone. All performance measures are found by comparing the detected compression rate from the smartphone app with the reference rate from the *Resusci Anne* manikin. All tests were performed with an audible metronome running in the background to be sure to target the compression rates at the appropriate levels. The smartphone was lying on the floor next to the test manikin, along with a tablet used to record videos of the tests from approximately the same viewpoint as the phone. These videos could later be analyzed to see what was going on in the test. All tests were performed at Laerdal Medical.^5^ Average error and standard deviation was calculated over the timespan of the individual tests. There will be a given lag (approx. 0.5 s) between the reference rate and the detected compression rate since all compression rate methods has to be performed over a time window. The results have been corrected for this lag to make the comparison as fair as possible.

#### Experiment 1—different target frequencies

**Fig. 6:**
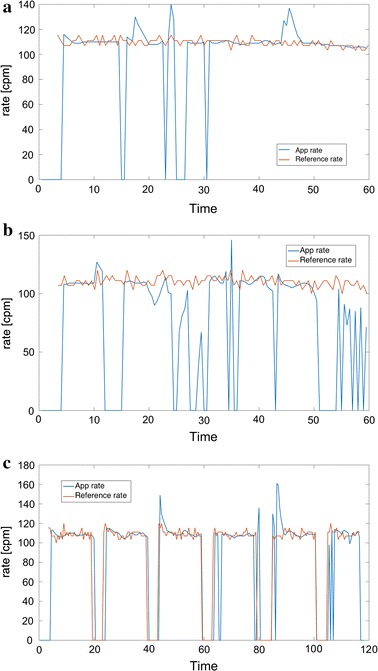
Three test examples, time in seconds, and compression rate in cpm. *Blue line* corresponds to compression rate from smartphone app, and *red line* corresponds to reference rate recorded from the manikin.** a** Test person 2 (long loose hair), target compression rate 110 cpm. $\bar{E}$ = 11.3, P = 80.2.** b** Test person 5 (long loose hair), bad phone placement AND background disturbances. Target rate = 110 cpm. $\bar{E}$ = 34.8, P = 49.4.** c** Test person 5 (long loose hair), CPR 30:2. $\bar{E}$ = 7.9, P = 82.7

An experiment was done without background disturbances and with target compression rates at both 60 and 110 cpm for all test persons. Each test is one minute long, and an example is seen in Fig. 6a. CPR according to the guidelines, is recommended to follow a 30:2 pattern, i.e. 30 compressions followed by two rescue breaths when performed by a single bystander, and the recommended compression rate is 110 cpm. Thus in this experiment without disturbances a 30:2 pattern with target compression rate at 110 cpm is also tested. These tests are performed over approximately 2 min for all test persons, and an example is seen in Fig. 6c.

#### Experiment 2—different disturbances

The algorithm was tested under different situations designed to test the algorithms performance in situations that involved more than just consecutive compressions. The situations tested were the following:

- Background disturbances by other people being visible in the video frame, possibly moving around.
- Suboptimal placement of the phone so that only a fraction of the video frame captures the bystander.
- A combination of suboptimal phone placement and background disturbances.
- CPR 30:2 in combination with background disturbances.
- Varying background illumination.
- Random movement of a person *not* performing compressions.

The amount of illumination or the disturbances is not controlled or measured in a quantifiable way in this experiment. The illumination test is done by changing from (i) indoor light in ceiling and daylight through windows, (ii) indoor light of, just some daylight through partly occluded windows. During optimal placement, the head of the bystander is approximately in the middle of the camera frame. For the suboptimal placement, the phone is placed so that only a small part of the head or shoulder is visible in an edge of the camera frame. The background disturbance is added by letting other people be visible in the videoframe, moving and walking around in the background.

The target compression rate was 110 cpm, except in the random movement test where there is no target rate. The duration of the CPR 30:2 tests are approximately 2 min, whereas all the other tests are approximately 1 min. In the random movement test, the test person performed other possible activities a bystander might be doing, i.e. checking for pulse or breath, looking around (for help), sitting by the manikin but not performing compression, thus the reference compression rate is 0.

## Results

**Fig. 7:**
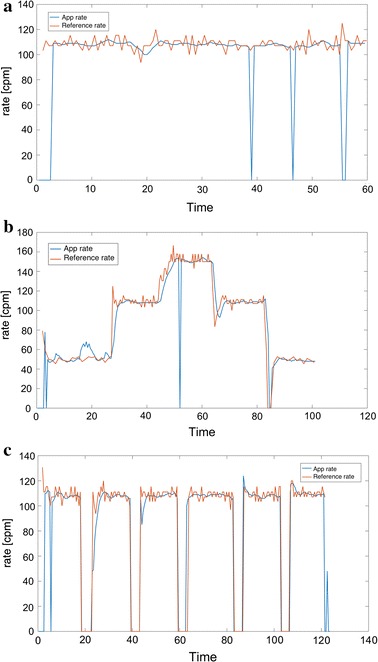
Three test examples, time in seconds, and compression rate in cpm. *Blue line* corresponds to compression rate from smartphone app, and *red line* corresponds to reference rate recorded from the manikin.** a** Test person 6 (medium length hair), target compression rate 110 cpm. $\bar{E}$ = 6.4, P = 90.4.** b** Test person 4 (short hair), different target compression rates. $\bar{E}$ = 5.0, P = 85.2.** c** Test person 3 (long hair in topknot, considered short hair), CPR 30:2. $\bar{E}$ = 3.3, P = 89.8

Some examples of tests with different test persons are seen in Fig. 7. The blue line shows the estimated compression rate as the output of the proposed system implemented as a smartphone app, and the red lines show the reference compression rate recorded by the manikin. The figure shows one example of a test where the target compression rate is held constant, another where the target rate varies, and a third plot showing a CPR 30:2 test. To evaluate the quality of the proposed system, two measures are defined. Let $x_{app}(n)$ correspond to the time series signal giving the estimated compression rate at a given time, i.e. the blue lines in Fig. 7. Let $x_{manikin}(n)$ denote the compression rate recorded from the manikin as a function of time, corresponding to the red signals. The average error, $\bar{E}$ [cpm], is found as:

14 $$\begin{aligned} \bar{E}=\frac{1}{N}\sum _{n=1}^{N} E(n)=\frac{1}{N}\sum _{n=0}^{N-1} |x_{app}(n)-x_{manikin}(n)| \end{aligned}$$

where *N* is the number of samples in the test, corresponding to:

15 $$\begin{aligned} N= \text{ TestD }[s]\times \frac{\text{ video } \text{ rate } [fps]}{15 \; \text{frames}/\text{sample}} \end{aligned}$$

where TestD is the duration of the test in seconds. 15 corresponds to the choice of $L=15$ in Eq. 3. The reported average error, $\mu _{\bar{E}}=\text{ mean }(\bar{E})$ and $\sigma _{\bar{E}}=\text{ std }(\bar{E})$, is found as the mean and standard deviation of the $\bar{E}$ over the different tests (i.e. different test persons):

16 $$\begin{aligned} \mu _{\bar{E}}=mean(\bar{E})=\,& {} \frac{1}{No}\sum _{i=1}^{No}\bar{E}_{i}, \end{aligned}$$

17 $$\begin{aligned} \sigma _{\bar{E}}=std(\bar{E})=\,& {} \sqrt{ \frac{1}{No-1}\sum _{i=1}^{No}(\bar{E}_{i}-\mu _{\bar{E}})^{2} } \end{aligned}$$

where $\bar{E}_{i}$ is the average error according to Eq. 14 for test number *i*, and No is the number of tests.

The performance, *P*, is defined as percentage of time where the difference between the algorithm rate and manikin rate is less than 10 cpm:

18 $$\begin{aligned} P=\frac{100}{N}\sum _{n=0}^{N-1} s(n). \end{aligned}$$

*s*(*n*) is an indicator function defining if the app rate is *close enough* to the reference rate from the manikin, where this is defined to be a difference less than 10 cpm:

19 $$\begin{aligned} s(n) = \left\{ \begin{array}{l l} 1, \quad &{} |x_{app}(n)-x_{manikin}(n)| < 10 \; \text{ cpm }\\ 0, \quad &{} otherwise \end{array} \right. \end{aligned}$$

The reported average performance, $\mu _{P}=\text{ mean }(P)$ and $\sigma _{P}=\text{ std }(P)$, is found as the mean and standard deviation of the *P* over the different tests (i.e. different test persons):

20 $$\begin{aligned} \mu _{P}=mean(P)=\, & {} \frac{1}{No}\sum _{i=1}^{No} P_{i}, \end{aligned}$$

21 $$\begin{aligned} \sigma _{P}=std(P)=\,& {} \sqrt{ \frac{1}{No-1}\sum _{i=1}^{No}(P_{i}-\mu _{P})^{2} } \end{aligned}$$

where $P_{i}$ is the performance measure according to Eqs. 16 and 17 for test number *i*.

### Experiment 1—different target frequencies

The results of experiment 1 are shown in Tables 2 and 3. For a target rate of the recommended 110 cpm, the main results reported as $\text{ mean }(\bar{E}) ( \text{ std }(\bar{E}))$ [cpm] is 3.6 (0.77) for short hair, 6.8 (5.9) including medium length hair, and 8.7 (6.0) including long hair bystanders.

**Table 2:** Average error [cpm], $\mu _{\bar{E}}$ with $\sigma _{\bar{E}}$ in parentheses, for the different target rates and CPR 30:2 for groups of test persons

Test	Short hair/topknot	Short + med. hair	All test persons	Long loose hair
60 cpm	3.9 (1.77)	4.6 (1.6)	10.0 (11.0)	13.5 (3.1)
*110 cpm*	*3.6 (0.77)*	*6.8 (5.9)*	*8.7 (6.0)*	*23.5 (14.1)*
*CPR 30:2*	*3.9 (1.55)*	*13.4 (13.5)*	*18.6 (19.6)*	*31.5 (33.4)*

**Table 3:** Performance, $\mu _{P}$ and $\sigma _{P}$ where *P* is defined in Eq. 18, for the different target rates and CPR 30:2 for groups of test persons

Test	Short hair/topknot	Short + med. hair	All test persons	Long loose hair
60 cpm	87 (11)	86 (9.1)	68 (33)	25.1 (33.5)
*110 cpm*	*94 (2.8)*	*87 (13)*	*86 (11)*	*81.7 (2.1)*
*CPR 30:2*	*87 (3.4)*	*74 (19)*	*69 (24)*	*55.6 (38.3)*

*P* gives the % of time throughout the test where the detected rate is within a ±10 cpm limit compared to the recorded true compression rate

### Experiment 2—different disturbances

From preliminary off-line experiments and use of the app in real-time visual tests, as well as in the presented experiments, we have seen that long and loose hair is the disturbance with most impact on the result measures. Therefore the results are presented on groups according to hair-length. The results of experiment 2 are shown in Tables 4 and 5. Table 5 depicts the % of time that the algorithm detects a rate that corresponds to the recorded true rate within a limit of ±10 cpm. It can be observed that even with disturbances the algorithm provides a correct (±10 cpm) compression rate between 73 and 97 % of the time for short hair (or topknot) bystanders and between 66 and 92 % of the time when including medium haired bystanders. The worst results is combining CPR 30:2 pattern with disturbances in the form of another person walking around in the video background.

**Table 4:** Average error [cpm], $\mu _{\bar{E}}$ with $\sigma _{\bar{E}}\,$in paranthesis, for different disturbances for groups of test persons

Disturbances	Short hair/hair up	Short + medium hair	Short, medium + long hair
Backgr. dist.	5.1 (0.43)	8.2 (4.2)	18.4 (20.8)
Bad pos.	3.8 (2.65)	4.3 (2.0)	17.1 (29.1)
Dist + bas pos.	7.5 (2.77)	12.5 (9.0)	24.6 (24.8)
30:2 + dist.	18.5 (5.25)	23.6 (7.9)	28.6 (13.7)
Var. light	2.9 (0.96)	12.2 (16.8)	15.0 (17.7)
Other activities	38.1 (13.6)	26.7 (18.7)	28.5 (18.3)

The target rate is 110 cpm, except in the “other activities” test where there is no target compression rate

**Table 5:** Performance, $\mu _{P}$ and $\sigma _{P}$ where *P* is defined in Eq. 18, for different disturbances for groups of test persons

Disturbances	Short hair/hair up	Short + medium hair	Short, medium + long hair
Backgr. dist.	85 (4.6)	82 (8.9)	71 (21)
Bad pos.	91 (10)	92 (7.2)	77 (34)
Dist + bas pos.	80 (10)	76 (14)	63 (26)
30:2 + dist.	73 (6.3)	66 (11)	59 (19)
Var. light	97 (1.3)	84 (22)	80 (21)
Other activities	53 (19)	67 (23)	62 (24)

*P* gives the % of time throughout the test where the detected rate is within a ±10 cpm limit compared to the recorded true compression rate. The target rate is 110 cpm, except in the “other activities” test where there is no target compression rate

## Discussion

In all experiments a metronome was used to target a compression rate. Our reference for calculated error and performance measurements is the recorded chest compression rate from the manikin, i.e *not* the target rate. A metronome could easily be built in the app to provide a target rate of 110 cpm. to achieve sound guidance as demonstrated by Park et al. [17].

### Discussion, experiment 1

For bystanders with short hair, the algorithm performs very well. Including test persons with medium length hair and thereafter with long hair gives a drop in the performance, but still for compression at 110 cpm (recommended target rate) the performance is very good. For CPR 30:2 pattern, the performance drop is more significant from short hair, to including long and medium hair. A possible explanation is the random movement of long hair when the bystander changes between providing chest compression and giving breaths.

**Fig. 8:**
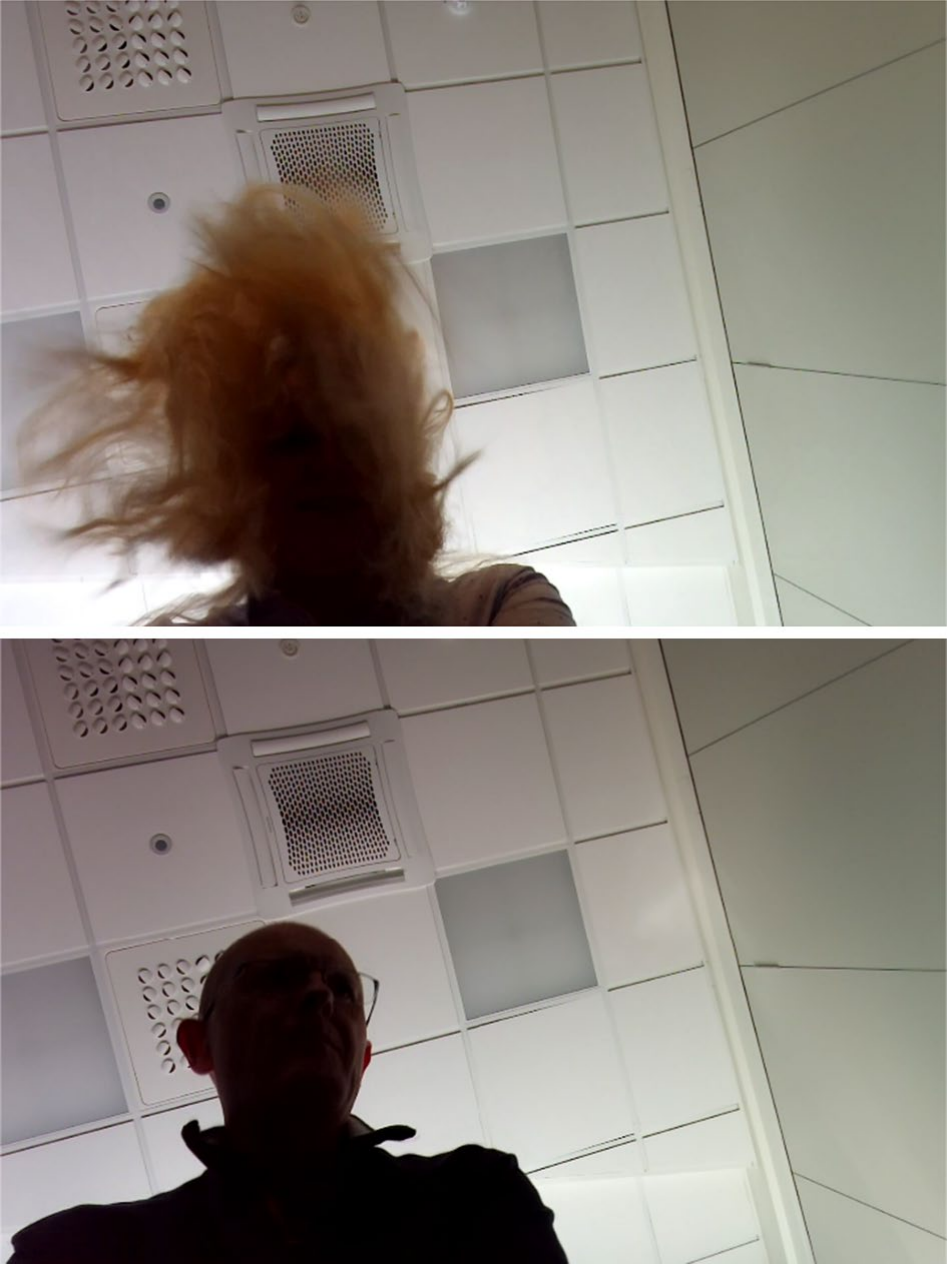
Examples of video frames during compression. *Top* bystander with long hair, *bottom* bystander with short hair

Examples of captured video frames during tests of person with long hair and another with short hair are seen in Fig. 8, indicating why long hair can be a problem for the detection algorithm. Long hair will move somehow more chaotic than short hair, causing a lot of different frequencies in the FFT of the difference signal. Thus the frequency corresponding to the actual compression rate might be hidden, and another frequency might mistakenly be detected and displayed as the compression rate. Figure 6 shows that in some cases of long and loose hair, like in (a) and (c), the detected rate is correct in large parts of the time, but in the example (b) the detected rate is changing all the time. This is a topic of further research, where we will try to improve the detected rate for long and loose hair. It is also possible to incorporate feedback on when the detected rate is trustworthy or not. A trustworthy feedback signal would usually quickly become quite stable, with the exception of the occasional missed detection, as seen as blue line drops in Fig. 7. The missed detection rate time points is seen as a drop to zero in a very short time interval.

### Discussion, experiment 2

As seen from Table 4, the algorithm handles most disturbances in the background well. For short hair it is not important where the phone is placed as long as something of the bystander is visible, and background disturbances and variable lights gives only very small degradations of the algorithm performance. For long hair bystanders the disturbances had somewhat more negative effect. It was observed during testing that the dynamic ROI finder was an important part of the algorithm to make it robust to disturbances. Movements outside the ROI did not affect the reported compression rate at all (as expected). Small background movements inside the ROI seems to be handled well, for the most part it did not affect the detected compression rate. However, large movements inside the ROI would in some cases cause drops or spikes in the detected compression rate, depending on how large the movements were in relation to the compression movements, and how they interfered with each other.

The CPR 30:2 with disturbances, however, shows a more significant degraded performance than the other disturbances. During this test it became clear that it was possible for the disturbance person in the background to firstly infiltrate and thereafter take over the ROI when the test person were performing rescue breaths. In this situation, the results are unpredictable. If the bystander performing compressions ends up being outside the ROI, none of the compressions will be registered. If the compressions are inside the ROI, the compressions might be picked up, depending on how large the compression movements are in comparison with the background movements. If the test person got back into the ROI, or if the person in the background disappeared out of the video frame, the correct rate was quickly recovered. This might, however, be a rare situation since when there are multiple bystanders it is recommended that one of them is doing compression and another rescue breaths.

The *other activities* test can be seen as a specificity test, and it performs apparently significantly worse than the other disturbance tests. However, the “true signal” is here always considered to be 0 [cpm], thus when the performance is tested is measuring in practice how often the algorithm produces 0, since $\{-10, 10\}$ is not within valid compression rates. The compression rate displayed to the dispatcher will usually vary a lot in this situation, and should make the dispatcher questioning if there are compressions being performed. However we consider to improve the specificity as well as working on improving the algorithm for when the bystander has long hair, to be the two main goals for our future research.

## Conclusions

A smartphone app for estimating compression rate during chest compression and CPR using the built-in camera, and performing all calculations in real time, has been proposed, implemented and tested. For target compression rates at around 100–110 cpm, which is the desired compression rate during CPR, the algorithm performs very well even with variations in illumination, disturbances in the background, CPR 30:2, and suboptimally placed phone. However, there is one factor that influences a lot, and that is if the bystander has long and loose hair. In that case, the algorithm is far less reliable due to the noise from the fluttering hair. In future work we hope to improve the algorithm for the case of the bystander having long and loose hair, as well as improve the specificity to detect the absence of repetetive compression even if there are a lot of movements in the video frame. In future research we will try to add disturbances in a quantifiable and consistent manner, and we will also include outdoor testing.

## Abbreviations

CPR: cardiopulmonary resuscitation
GPS: global positioning system
FFT: fast fourier transform
OHCA: out-of-hospital cardiac arrest
AED: automated external defibrillator
ERC: European resuscitation council
ROI: region of interest
cpm: compressions pr. minute
fps: frames per second

## Footnotes

1 URL: http://www.heart.org/idc/groups/heartpublic/@wcm/@adv/documents/downloadable/ucm_461797.pdf.

2 URL: https://www.itunes.apple.com/no/app/hjelp-113-gps/id363739748?l=no&mt=8.

3 URL: https://www.play.google.com/store/apps/details?id=com.pocketcpr.poccpr.

4 URL: http://www.laerdal.com/gb/ResusciAnne.

5 URL: http://www.laerdal.com/.
